# Ultrasensitive Quantification of MUC16 Antigen/Amine-Terminated Aptamer Interaction by Surface Plasmon Resonance: Kinetic and Thermodynamic Studies

**DOI:** 10.34172/apb.2024.028

**Published:** 2024-01-13

**Authors:** Shahnam Valizadeh Shahbazlou, Somayeh Vandghanooni, Bahareh Dabirmanesh, Morteza Eskandani, Sadegh Hasannia

**Affiliations:** ^1^Department of Biochemistry, Faculty of Biological Sciences, Tarbiat Modares University, Tehran, Iran.; ^2^Hematology and Oncology Research Center, Tabriz University of Medical Sciences, Tabriz, Iran.; ^3^Research Center for Pharmaceutical Nanotechnology, Biomedicine Institute, Tabriz University of Medical Sciences, Tabriz, Iran.

**Keywords:** Aptasensor, MUC16, Ovarian cancer, Real-time, Surface plasmon resonance

## Abstract

**Purpose:**

MUC16 is a commonly employed biomarker to identify and predict ovarian cancer (OC). Precise measurement of MUC16 levels is essential for the accurate diagnosis, prediction, and management of OC. This research seeks to introduce a new surface plasmon resonance (SPR) biosensor design that utilizes aptamer-based technology to enable the sensitive and real-time detection of MUC16.

**Methods:**

In this study, the sensor chip was immobilized with an anti-MUC16 aptamer (Ap) by utilizing 11-mercaptoundecanoic acid (MUA) as a linker to attach the amine-terminated Ap to the chip using EDC/NHS chemistry.

**Results:**

The results indicated that the newly created aptasensor had a detection limit of 0.03 U/mL for MUC16 concentration, with a linear range of 0.09 to 0.27 U/mL. The findings demonstrate good precision and accuracy (<15%) for each MUC16 concentration, with recoveries ranging from 93% to 96%. Additionally, the aptasensor exhibited high selectivity, good repeatability, stability, and applicability in real human serum samples, indicating its potential as a valuable tool for the diagnosis and treatment of OC.

**Conclusion:**

According to the outcomes, the designed aptasensor exhibited acceptable specificity to detect the CA125 antigen and could be utilized for the serum detection of target antigen by SPR method.

## Introduction

 Carbohydrate antigen 125 (CA125) or MUC16 (the gene encoding MUC16) is a cell surface glycoprotein^1^ that is commonly used as a tumor marker for the diagnosis and prognosis of ovarian cancer (OC).^2^ OC is the seventh most common cancer and the eighth worldwide main cause of mortality among women.^3,4^ MUC16 effectiveness in diagnosing early stages of OC is limited due to its low sensitivity. This is primarily due to the fact that MUC16 levels can be elevated in other physiological or pathological conditions as well. For instance, MUC16 levels may increase during menstruation, pregnancy, or in cases of peritoneal inflammatory diseases. These factors can lead to false positives and make it difficult to accurately diagnose OC using MUC16 levels alone.^2,5-8^ In addition, because of the low sensitivity and specificity of the MUC16 using commercial techniques, it is evaluated in combination with other biomarkers as an algorithm.^9,10^ However, the level of MUC16 is estimated in the follow-up monitoring after surgery and chemotherapy OC. The normal range of MUC16, as cancer surveillance, is lower than 35 U/mL^11^ and increased levels of MUC16 (more than 65 U/mL) are associated with a lower 5-year survival rate.^12,13^ The five-year survival rate is less than 30% for more than 70% of patients at an advanced stage. However, it is up to 90% and 70% for patients diagnosed with stage I and stage II, respectively.^14^ OC is hardly detected at an early stage because of the lack of specific screening tools and vague symptoms. Early detection of OC using different serum biomarkers is associated with improved clinical outcomes.^15^ The commercial screening techniques (*e.g.,* electrochemiluminescence [ECL] and enzyme-linked immunosorbent assay [ELISA]) are unable to detect OC at an early stage.^16^ Recently, surface plasmon resonance (SPR) has been developed as a highly efficient label-free technique with numerous advantages (*e.g.,* direct, highest reproducibility, and real-time detection).^17,18^ This optical technique is developed based on the SPR phenomenon and measures the refractive index (RI) changes occurring at the thin metal layers surface (*e.g.,* gold and silver films) caused by biomolecular interactions.^19^ Various investigators tried to develop different SPR-based biosensors to quantify biomolecular interactions.^20^ Hsieh et al developed an aptasensor based on SPR for detecting the Epstein–Barr virus with a limit of detection (LOD) of 10 pg/mL.^21^ Gyurcsanyi et al reported an SPR biosensor based on Ap for the detection of immunoglobin E (IgE) with a detection range from 0.156–40 µM.^22^ Chen et al designed an SPR biosensor to determine exosomes from prostate cancer cells. The linear range achieved was 1 × 10^5^ to 1 × 10^7^ particles/mL with an LOD of 1 × 10^5^ particles/mL.^23^ A study used SPR to measure MUC16 by immobilizing a rabbit anti-human MUC16 antibody and the LOD was found to be 0.66 U/mL. The linear range of values ranged from 2.2 to 150 U/mL.^16^ In a similar study Siriwan et al quantified MUC16 with LOD of 0.1 U/mL in the linearity of 0.1–40 U/mL.^24^ SPR biosensors have several advantages over conventional diagnostics. These include increased sensitivity and selectivity, reusable sensor chips, real-time monitoring, the ability to analyze several targets in parallel, label-free detection, and the ability to perform point-of-care diagnostics.^25^ Compared to carbon-based ink fabrication, SPR-based aptasensors provide higher sensitivity for differentiating and quantifying the analyte at concentrations ranging from picomolar to femtomolar. The sensors also offer reusable chips, short assay time, and increased stability.^26^ In our previous investigation, we established an SPR-based aptasensor for the detection of MUC16 by immobilization of biotinylated-Aps with streptavidin-biotin interaction.^27^ It seems that some complications lead to non-specific binding between biotin and target antigen which may cause potentially false-positive results. The Arg-Tyr-Asp (RYD) sequence in streptavidin resembles the Arg-Gly-Asp (RGD) sequence. The triple RYD sequence is not part of the streptavidin-biotin binding site and causes the background in the test result. Hence, it can be the binding site of some serum proteins. Consequently, nonspecific binding with components other than the MUC16 antigen may lead to false positive results.^28,29^ Moreover, although this immobilization method is easy to perform, low ionic strength conditions can disassociate the streptavidin-biotin complex.^30^ Therefore, we investigated the SPR-based aptasensor with *N*-hydroxysuccinimide (NHS) and N-Ethyl-N’-(3-dimethylaminopropyl)-carbodiimide (EDC) activation chemistry in this work.

 Our study aimed to develop a highly sensitive and selective Ap-based SPR biosensor for detecting MUC16 tumor markers in blood serum by using DNA Ap as the targeting ligand of the MUC16 biomarker. In this study, amine-terminated MUC16 Ap was immobilized on a gold chip surface using an 11-mercaptoundecanoic acid linker with EDC/NHS chemistry. We optimized the temperature and pH of the flow buffer and calculated the equilibrium dissociation constant (K_D_) as well as other bioanalytical method validation elements such as LOD, selectivity, specificity, accuracy, precision, and recovery. This study’s novelty lies in the significant improvement of validation parameters. Finally, to ensure the accuracy and reliability of the developed sensor, the obtained results were compared with those from commercial screening tests (ELISA). The purpose of this comparison was to verify that the sensor is capable of detecting and measuring the MUC16 biomarker in human serum samples with high sensitivity and reliability, while operating under optimal conditions. The study aimed to ensure that the sensor’s performance is accurate and dependable in detecting this particular biomarker.

## Materials

 The bare chips for the SPR sensor were acquired from Bionavis Company, located in the Tampere region of Finland. Phosphate buffered saline (PBS), 11-mercaptoundecanoic acid (MUA), NHS, EDC, carcinoembryonic antigen (CEA), bovine serum albumin (BSA), prostate-specific antigen (PSA), and cancer antigen 19-9 (CA19-9) were purchased from Sigma-Aldrich (St. Louis, Missouri, USA). The 3ʹ-[C6 Amine] MUC16 Ap: 5’- CTC ACT ATA GGG AGA CAA GAA TAA ACG CTC AA-3ʹ (32bp) were obtained from Bioneer (Bioneer Inc, Korea )^31^ and reconstituted in nuclease-free water. The MUC16 antigen was purchased from Monobind (Monobind Inc, USA). All the other chemicals used were of analytical grade.

###  SPR measurement 

 The SPR measurement was performed using a Multi-Parametric SPR device, specifically the MP-SPR Navi 210A model, manufactured by BioNavis Ltd in the Tampere region of Finland. The device utilized two distinct flow channels during the experiment. The device was equipped with a cohesive peristaltic pump with 100 μL sampler loops for the sample transport. To immobilize the anti-MUC16 Ap, a gold chip consisting of a BK-7 glass plate (240 mm^2^) coated with a thick layer of gold (50 nm) was employed. This configuration was chosen as the substrate for the immobilization process. The same refractive index (n= 1.518) oil was utilized for adhering of glass side of the chip to the device prism. The SPR signal is expressed in resonance or response unit (RU). The entire flow path was purged with run buffer prior to analysis (50 mL/min; 15 minutes) and the SPR measurements were done at a flow rate of 10 μL/min with 20 μL sample volume during the experiment. The interaction of different concentrations of MUC16 antigen and Ap-decorated surface was evaluated by flowing MUC16 over the sensor surface at the constant wavelength angle (670 nm). The kinetic analysis was conducted in phosphate buffer (PB) with varying pH levels (7.2, 4.2, and 8) at different temperatures (25 °C, 37 °C, and 42 °C). The data was obtained through the use of SPR Navi^TM^ data viewer software. Kinetic parameters regarding MUC16 binding to the Ap were then calculated using Trace Drawer^TM^ for SPR Navi^TM^.

###  Fabrication of the chip surface

 To prepare the gold chip for analysis, it was boiled in a solution containing 30% hydrogen peroxide, 30% ammonia (NH_4_OH), and Milli-Q-water in a ratio of 1:1:5. The mixture was heated to 95 °C for 10 minutes. The chip was rinsed three times with Milli-Q-water and three times with ethanol solution being dried with N_2_ gas. To the formation of the self-assembly monolayer (SAM), the gold chips were submerged in MUA (200µL; 2 mM) solution at room temperature (RT; 24 ± 2 °C) for 24 h. Following the preparation of the SAM chips, they were washed three times with absolute ethanol and dried using N2 gas. Once the rinsing and drying process was complete, the SAM chips were inserted into the holding block of the SPR device for further analysis.

###  Immobilization of MUC16 Ap onto the SAM chip surface

 Further cleaning of the sensor surface was performed by running NaCl (2 M; 75 μL; 25 μL/min) and NaOH (0.1 M; 75 μL; 25 μL/min) in a ratio of 1:1 (3 min). Then solution of EDC (150 μL; 0.5 M) and NHS (150 μL; 0.1 M) in a ratio of 1:1 (3 minutes) were used for the activation of the carboxylic acid groups on the SAM. Before use, Aps should be folded into their tertiary structure for optimal binding. To carry out this procedure, the Ap was initially suspended in water that was free of nucleases, and then incubated at room temperature for a period of 30 minutes. The resulting Ap solution was subsequently divided into smaller portions, or aliquots, and stored at a temperature of -20 °C for future use. To achieve its working concentration (200 μL; 20 pmoles/µL), the Ap was reconstituted in a folding buffer containing PBS buffer and 1 mM MgCl2. To renature the Aps, a DNA engine thermocycler (Bio-Rad, Singapore) was used. The process involved heating the Aps to 85 °C for 10 minutes, followed by cooling down at a rate of 0.5 °C/s until it reached 4 °C. Then, the Ap solution was injected into channels at a flow rate of 20 μL/min. The online response was used to characterize Ap immobilization. Finally, BSA [0.1% (w/v); 60 µL] was injected for the blocking of remaining unreacted groups.

###  MUC16 assay and real sample analysis

 To optimize the performance of the developed aptasensor, the pH of the flow buffer and temperature were varied. During each cycle, the flow rate was set to 10 μL/min. A dissociation time of 120s followed by 120s association time was applied for the assay. Different concentrations of MUC16 (0.09-0.27 U/mL) were prepared in phosphate buffer at varying pH levels (pH 7.2, pH 4.2, and pH 8). A calibration curve was generated by plotting the relative signal against the concentration of MUC16. To confirm the presence of a matrix effect, a serum sample was procured from a group of healthy individuals at Shahid Ghazi hospital, located in Tabriz, Iran. The study was conducted in accordance with the principles outlined in the Declaration of Helsinki, and informed consent was obtained from a healthy volunteer who participated in the study.^32^ To prepare spiked samples, different concentrations of MUC16 were added to diluted human serum in phosphate buffer (1% v/v), then the calibration curve was plotted. The SPR measurements were performed in the optimum conditions (pH 7.2 and 25 °C) same as the mentioned procedures. The signal recorded represents the difference between the values obtained in channel 1 and channel 2.

###  Kinetic parameters

 To determine the kinetic parameter, various concentrations of MUC16 (ranging from 0.09 to 0.27 U/mL) were injected into different buffer environments at a flow rate of 10 μL/min for 2 minutes. The affinity parameters for the interaction between Ap and MUC16 were analyzed using the TraceDrawer^TM^ software. The rate constants for association (*k*_on_) and dissociation (*k*_off_) were determined, along with the equilibrium association constant K_D_ (*k*_off_/*k*_on_). These constants represent key parameters in characterizing the binding interactions between the antigen and Aps. The obtained data was fitted to a 1:1 interaction model, denoted as (A + B⇌AB). A represents the injected antigen (MUC16), B represents the immobilized receptor (Ap), and AB represents the antigen-receptor complex (MUC16-Ap).

###  Thermodynamic analysis of MUC16/Ap interaction

 To investigate the temperature effect on MUC16/Ap binding, SPR experiments were conducted at different temperatures (298, 303, and 310 °K). The thermodynamic analysis was then performed using the van’t Hoff equation.

###  Validation data processing 

 To assess the method’s precision, accuracy, and recovery, triplicate measurements were investigated within the calibration curve range. To assess the precision and accuracy of the results, two metrics were employed. The first metric, percentage bias, was used to determine the difference between the actual and calculated concentrations of each sample. This allowed for an evaluation of the accuracy of the measurements taken. The second metric, relative standard deviation (RSD%), was utilized to express the precision of the measurements. The RSD% metric demonstrated the level of agreement between repeated measurements taken over time.^33^ The recovery indicates the efficiency of the extraction method by comparing the ratio of the results obtained from low, medium, and high concentrations to the response of the pure standard.^34^ Repeatability reflects the precision under the same operating conditions over a short time interval (intra-assay) and is reported as the standard deviation (SD) of a series of measurements.^35^ Stability was determined by replicate measurement of the spiked sample solution and expressed as a percentage of stability and could be calculated by % stability (ratio of mean response of stability samples/mean response of comparison samples).^34^ In accordance with the guidelines established by the International Conference on Harmonization (ICH), the LOD and limit of quantification (LOQ) were determined using specific calculations. The LOD and LOQ were calculated based on the SD of the response obtained from the blank samples (which contained no antigen) and the slope of the calibration curve (s). Specifically, the LOD was determined as 3.3 times the standard deviation divided by the slope (3.3σ/s), while the LOQ was calculated as 10 times the standard deviation divided by the slope (10σ/s). These calculations allowed for the determination of the minimum detectable and quantifiable levels in the analysis.^36,37^ To investigate the selectivity of the Ap surface toward MUC16, we tested the aptasensor’s specificity towards various OC tumor markers, such as CA 19-9, PSA, and CEA. To assess the specificity of the aptasensor, its performance was evaluated in the presence of a mixture of interfering antigens. This test was conducted to determine how well the aptasensor could differentiate and selectively detect the target antigen in the presence of other potentially interfering substances. By subjecting the aptasensor to this test, its ability to accurately identify and respond to the target antigen in complex samples was evaluated.

###  The efficiency comparison of designed biosensor and commercial tests 

 To assess the effectiveness of the newly developed aptasensor, the detection of the MUC16 antigen was carried out in both spiked samples and standard samples. The results obtained from the aptasensor were then compared to those obtained from established diagnostic laboratory techniques such as ELISA and ECL. This comparison allowed for an evaluation of the aptasensor’s performance and its potential as a reliable alternative to existing diagnostic methods.

###  Statistical analyses

 The statistical analyses for this study were conducted using the SPSS software (Statistical Package for the Social Sciences, version 13.0, SPSS Inc, Chicago, Illinois, USA). The independent samples *t* test was performed to determine the comparison between triplicated standard and spike measurements. To compare the slopes of the calibration curves, the one-way ANOVA analysis with Tukey’s post hoc analyses was used. To assess significant differences between groups, *P* values were considered less than 0.05. TraceDrawer^TM^ software (Dag Hammarskjölds väg 36A, Science Park, 752 37 Uppsala, Sweden) was used for the determination of kinetic parameters.

## Preparation of the chip surface

**Figure 1:**
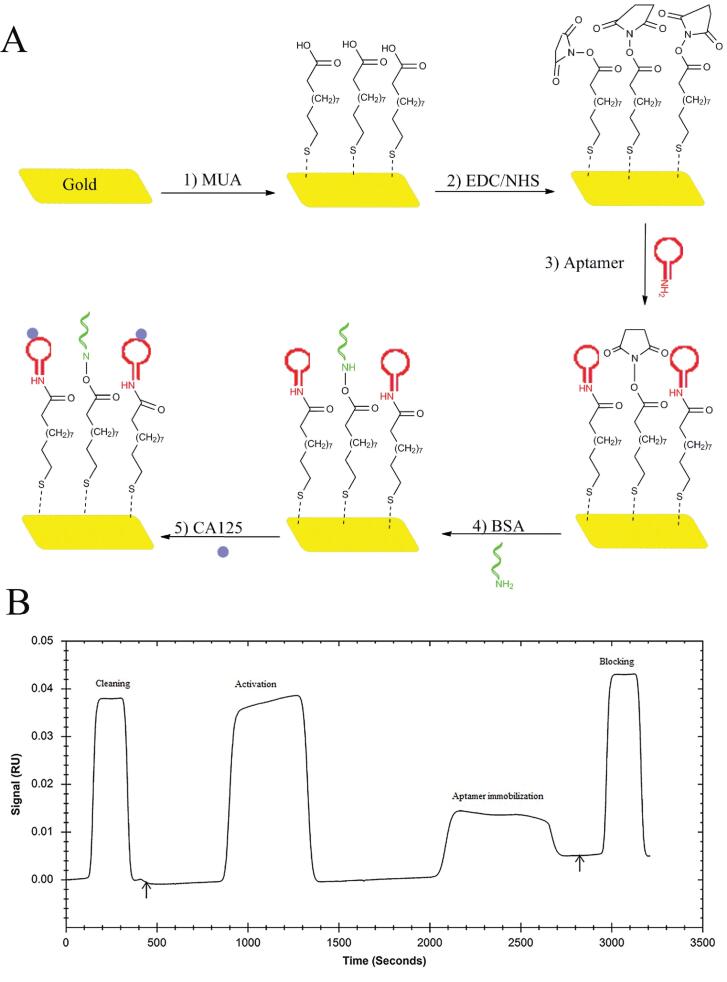
A) The scheme shows the development of the amine-terminated aptamer-based SPR biosensor for the detection of MUC16 tumor marker. B) SPR sensogram of immobilization of amine-terminated aptamer on the SAM surface of the gold chip. The aptamer immobilization was done through the injection of aptamer solution (200 µL; flow rate: 20 µL/min; flow buffer: 1 mM MgCl_2_, and 1X PBS), and the blocking of the nonspecific binding was done through the running of BSA [0.1% (w/v); 60 µL]

 The use of gold and silver nanoparticles (Ag NPs) and silver-based composite nanomaterials in the production of biosensors is a new and innovative approach to detecting biomolecules.^38,39^ However, studies have shown that Ag NPs or gold nanoparticles (Au NPs) can be toxic and potentially harmful to biorecognition molecules such as proteins, lipids, and DNA by inducing the production of reactive oxygen species (ROS).^40^ The cytotoxicity of Ag NPs and Au NPs depends on their various features, such as size, shape, surface charge, and coating material. On the other hand, Ag and Au in their molecular form, works as a catalyst and are toxic. To overcome this, we have chosen a gold chip as a basis for the aptasensor because Au is highly unreactive and chemically inert by nature, making bulk gold (bare gold chip) non-toxic.^41^ On a gold chip, Au-S bonds easily form, enabling the fast attachment of receptors via the SAM surface. In most cases, MUA is used to transform the functionalized monolayer directly. The availability of surface-bound carboxylic acid groups enables the covalent immobilization of aminated Aps through a range of diverse protocols. The presence of these carboxylic acid groups on the surface allows for the formation of stable covalent bonds with the aminated Aps.^42^ In this work, EDC/NHS chemistry was used to immobilize Aps on the sensor chip. The Aps molecules were introduced onto the surface and connected to the sensor chip using amine coupling. This process involved passing the Aps over the surface and facilitating their attachment through the amine coupling reaction. The schematic representation of this process, including the steps of immobilization and the subsequent interactions between the antigen and Aps, can be observed in Figure 1A. Figure 1B shows that the SPR signal increased by performing the cleaning step with NaCl (2 M) and NaOH (0.1 M) as well as by activation of SAM through injection of NHS/EDC (1:1). After activation the signal returned to the baseline. The SPR signal was increased as the Ap solution flowed on the surface of the sensor. After association time, the signal returned to baseline. The signal variation observed between the steady state and baseline measurements serves as an indicator of the immobilization of the Aps. Finally, the signal was increased and returned toward a steady state, indicating the sensor surface blocking by utilizing BSA (0.1% w/v).

###  Kinetics of MUC16binding to aptamer

**Figure 2:**
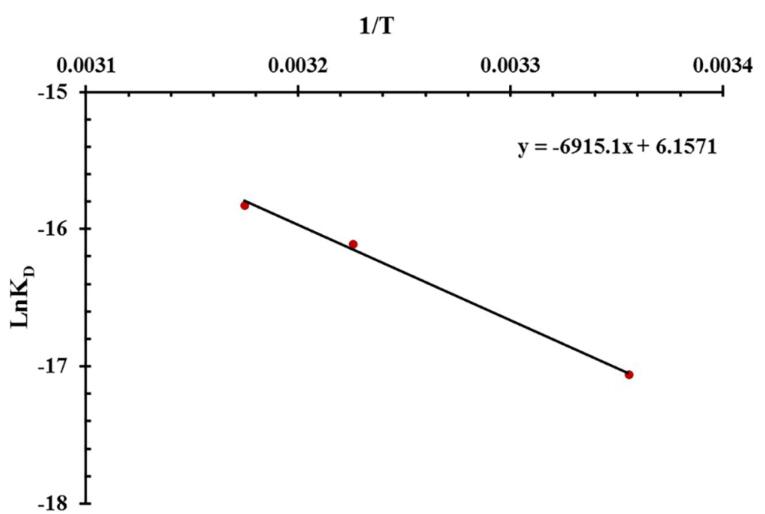
Van't Hoff plot for the MUC16/aptamer interaction in pH 7.2

Figure 2 displays the Van’t Hoff plot for the Ap interactions with different concentrations of MUC16. Under optimized pH 7.2 and 25°C conditions, the k_on_ and k_off_ values were 2.55 × 10^4^ M^-1^ s^-1^ and 1.01 × 10^-3^ s^-1^, respectively. The low K_D_ (*k*_off_/*k*_on_) value (3.9 × 10^-8^ M) indicates a high affinity of the MUC16-specific Ap to the MUC16 antigen. The thermodynamic parameters of the system were analyzed using the van’t Hoff equation ( $\ln\;K_{D}=-\frac{\Delta H}{RT}+\frac{\Delta S}{R}$ ), with the K_D_ value serving as a critical factor. Thermodynamic parameters including change in enthalpy (ΔH) and entropy values (ΔS) were evaluated to determine the type of interaction between the Ap and MUC16 antigen. ΔH and ΔS were obtained by plotting the lnK_D_ versus 1/T, where R and T indicate the universal gas constant and temperature, respectively (Figure 2). Then the Gibbs free energy (∆G) from the interaction was calculated by the standard Gibbs–Helmholtz equation (*∆G = ∆H-T∆S*).

 The type of interaction was determined based on the negative or positive values of ΔH and ΔS. Positive values of ΔS and ΔH frequently contribute to hydrophobic interaction whereas Van der Waals forces and hydrogen bonding derive from a negative ΔS value as well as a negative ΔH value.^43^ The electrostatic interaction originates from a positive ΔS value along with a negative ΔH value.^44^ Table 1 shows positive ΔH and ΔS values, indicating hydrophobic interaction under optimized pH 7.2 and 25 °C conditions. Since the ΔS value was positive, it can be envisioned that electrostatic interaction contributes to the binding process. Moreover, our results affirmed that a positive ΔG value indicates an endothermic binding process, which is dependent on enthalpy. The value of -TΔS was negative due to the value of ΔS was positive. Therefore, the value of ΔG small decreased and the K_D_ value increased when the temperature increased. In other words, the high temperature causes a lower affinity between Aps and MUC16 during SPR measurement. Additionally, high temperatures can affect the SPR system response by altering the surface plasmon conditions and the RI of the prism.^45^ Therefore, the optimum temperature for Ap and MUC16 interaction was 25 °C because the K_D_ is lower than compared to other temperatures (37 °C and 42 °C).

**Table 1:** Thermodynamic parameters of MUC16/aptamer interaction in pH 7.2 at different temperatures (298, 310, and 315 ^°^K)

**Temperatures (°k)**	**K**_D_	**∆H (J mol**^-1^**)**	**∆S (J mol**^-1^**)**	**∆G (J mol**^-1^** k**^-1^**)**
298	3.9 × 10^-8^	57492.14	51.19	42264.34
310	1.01 × 10^-7^			41651.14
315	1.34 × 10^-6^			41395.64

###  Optimization and assessment of the MUC16 biosensor

 The performance of the SPR-based aptasensor was optimized by altering the temperature and pH of the flow buffer. The results obtained here showed that MUC16 binds to the Ap irreversibly with a slow dissociation rate, as the relative response does not return to baseline. Depending on the pH of the flow buffer (PB) and isoelectric points of MUC16 (6.2–7.3), the surface charges on the antigens and Ap can be altered which may affect the capture rate as well as the efficiency of the aptasensor. Therefore, to evaluate the optimum conditions, experiments were performed in phosphate buffer at different pH values (7.2, 4.2, and 8) and various temperatures (25°C, 37°C, and 42°C). To generate a calibration curve for MUC16 concentration, various concentrations of the antigen were injected into the system in triplicate. The concentrations ranged from 0.09 to 0.27 U/mL. The relative response obtained from each concentration was then plotted against the corresponding MUC16 concentration. The relative response was recorded as the difference between the values obtained in channel 1 (working channel) and channel 2 (reference channel).

**Figure 3:**
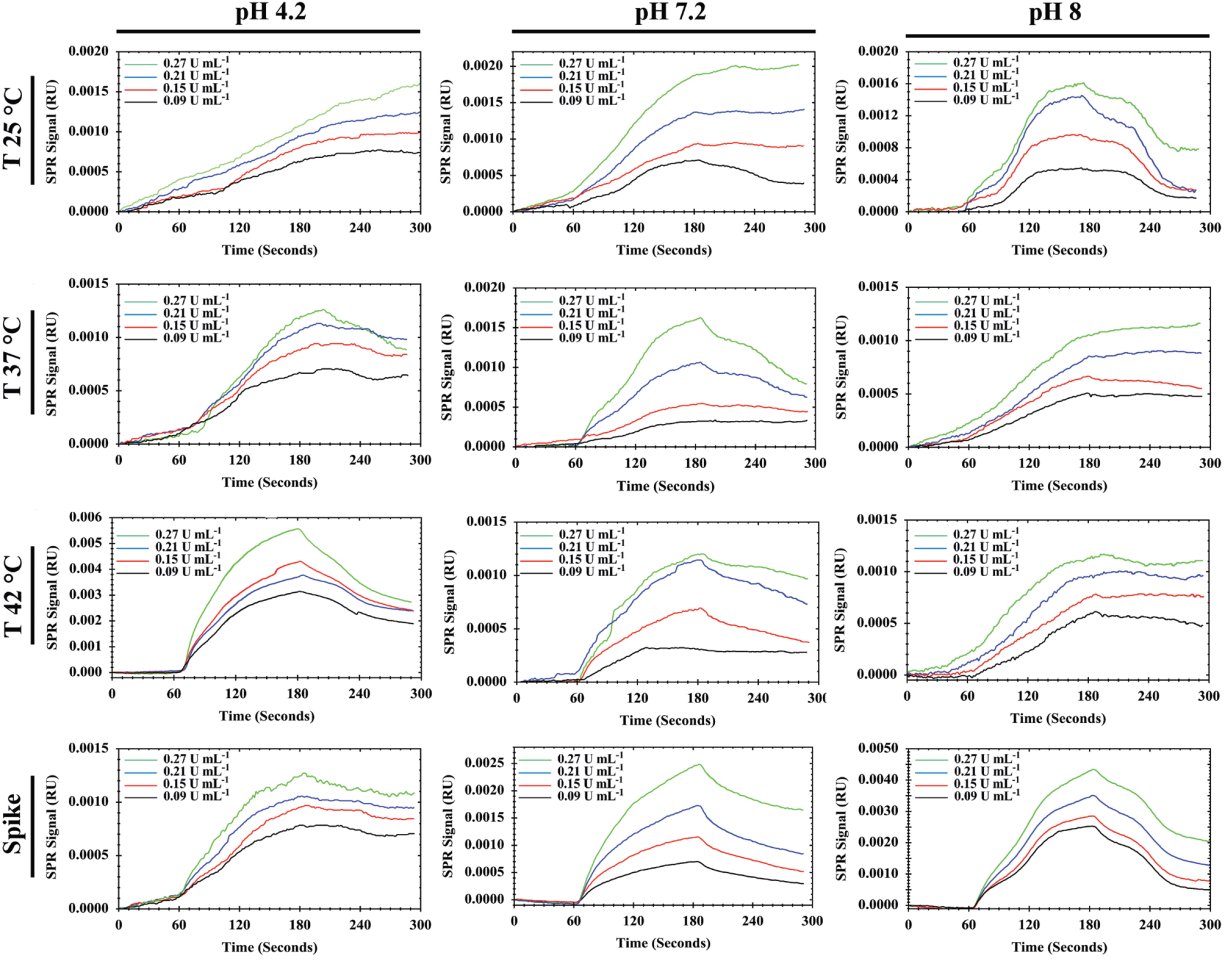
Sensograms of MUC16 detection (0.09–0.27 U/mL) in standard and spiked serum samples at different pHs (4.2, 7.2, and 8) and temperatures (25 °C, 37 °C, and 42 °C)

**Figure 4:**
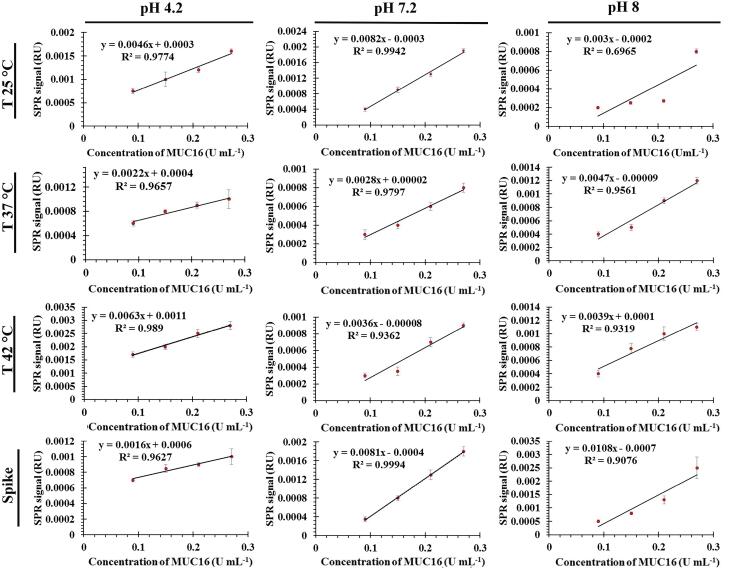
Calibration plots for the aptamer-based MUC16 aptasensor

 The sensogram and calibration curves for MUC16 and Ap interactions are shown in Figures 3 and 4.

 The increasing and decreasing tendency of the SPR signal in various conditions indicated that the binding (association and dissociation processes) of MUC16 to Ap could be influenced by flow buffer conditions (*e.g., *pH and temperature). Based on the linear regression equation (y = 0.00082x - 0.0003, R^2^ = 0.9942), the buffer (pH 7.2, 25 °C) was selected as the optimum condition. The corresponding sensogram shows that the drift of the baseline was close to zero and flat when the running buffer flowed over the sensor (the first 60 seconds). As the sample flowed over the Ap surface during association time (the period from 60 to 180 s), MUC16 bound with the Ap which resulted in the relatively increased signal which is directly affected by the concentration of the antigen. The slope of the association period suggests that higher concentrations of antigen would be injected. However, the results showed that the binding capacity of Aps to MUC16 antigen is highly dependent on the concentration of MUC16 antigen in which the higher concentration of antigen may cause decay on the surface of the sensor and interrupt biosensor signals. Besides, the association was free of mass transport limitation. During the dissociation period from 180 to 300 seconds, the signal did not return toward baseline because MUC16 interaction with Ap was strong and the buffer flow could not dissociate MUC16 and Ap binding. Furthermore, results show that the affinity constant K_A_ was low at alkaline and acidic pH mainly due to the high dissociation rate, indicating that the interaction could be decreased at high and low pH conditions. Therefore, it can be concluded that Aps and target antigen have the most opposite charges at the interface in pH 7.2.

 Temperature was also studied as one of the critical parameters affecting biomolecular interaction. Optical techniques such as SPR are highly dependent on temperature because they may influence the RI and corresponding signal.^46^ In this experiment, the temperature was gradually increased to prevent the formation of bubbles in the flow cell. However, the results indicate that increasing the temperature of the flow buffer leads to the formation of replicate curves and lower RU during SPR measurement. Figure 3 confirmed that the interaction between MUC16 and Aps enhanced by decreasing temperature. Previous studies developed antibodies-immobilized sensor surfaces to assess the interaction of MUC16 and antibodies and detect target MUC16 antigen in samples,^16,24^ while in the present study, the SPR-based aptasensor was fabricated for specific detection of MUC16 antigen. Aps have been successfully utilized for the development of different aptasensor platforms to detect target antigens due to their advantages compared to antibodies (*e.g.,* high specificity, stability to temperature, flexibility in structure, and ease of synthesis and modification).^47^ The LOD and LOQ of the assay were determined to be 0.03 U/mL (SD = 10%) and 0.09 U/mL (SD = 8%), respectively, within a linear range of 0.09 to 0.27 U/mL. Previous studies reported different types of biosensors for the detection of MUC16. Li et al developed an ECL biosensor labeled with Ru-AuNPs/graphene with a detection limit of 0.005 U/mL and a linear response range of 0.01–100 U/mL.^48^ Wu et al reported an ECL immunoassay with a LOD of 0.004 U/mLin the linearity of 0.01 U/mL-1000 U/mL.^49^ In another study, a fluorescence resonance energy transfer (FRET)-based biosensor developed with LOD of 0.5 fg/mL^1^ and a linear range of 1.0 fg/mL to 1.0 ng/mL of CA 125. AsTable 2 summarizes, numerous biosensors such as the antibody-based SPR biosensor and capacitive immunosensors system have reported different LOD.^24^ Results showed that the fabricated aptasensor in this research detects MUC16 antigen with a low LOD in comparison to the analytical performance of the other previously developed aptasensor.^16,50-52^ In addition, this SPR system can reduce substrate interference and improve validation parameters (*e.g., *accuracy, precision, and recovery). Compared to antibody-based biosensors, developed aptasensor offer easy establishment and operation, real-time monitoring, label-free detection, and faster assay times. Therefore, Ap-based SPR biosensor has great potential for the detection of target antigen at low concentrations.

**Table 2:** Comparison of different biosensor's efficiency for MUC16 biomarker detection

**Method**	**Linear range**	**LOD**	**Clinical sample**	**Ref.**
Electrochemical immunosensor	5-80 U/mL	1.45 U/mL	Serum	^51^
Electrochemiluminescence immunosensor	1 μU/mL-1 U/mL	0.1 μU/mL	Serum	^52^
SPR immunosensor	0.1-40 U/mL	0.1 U/mL	Serum	^24^
Capacitive system	0.05-40 U/mL	0.05 U/mL	Serum	^24^
Plasmon resonance scattering	1-80 U/mL	0.4 U/mL	PBS	^50^
SPR immunosensor	2.2-150 U/mL	0.66 U/mL	Serum	^16^
SPR aptasensor	10-100 U/mL	0.01 U/mL	Serum	^27^
SPR aptasensor	0.09-0.27 U/mL	0.03 U/mL	Serum	This work

###  Selectivity and specificity

**Figure 5:**
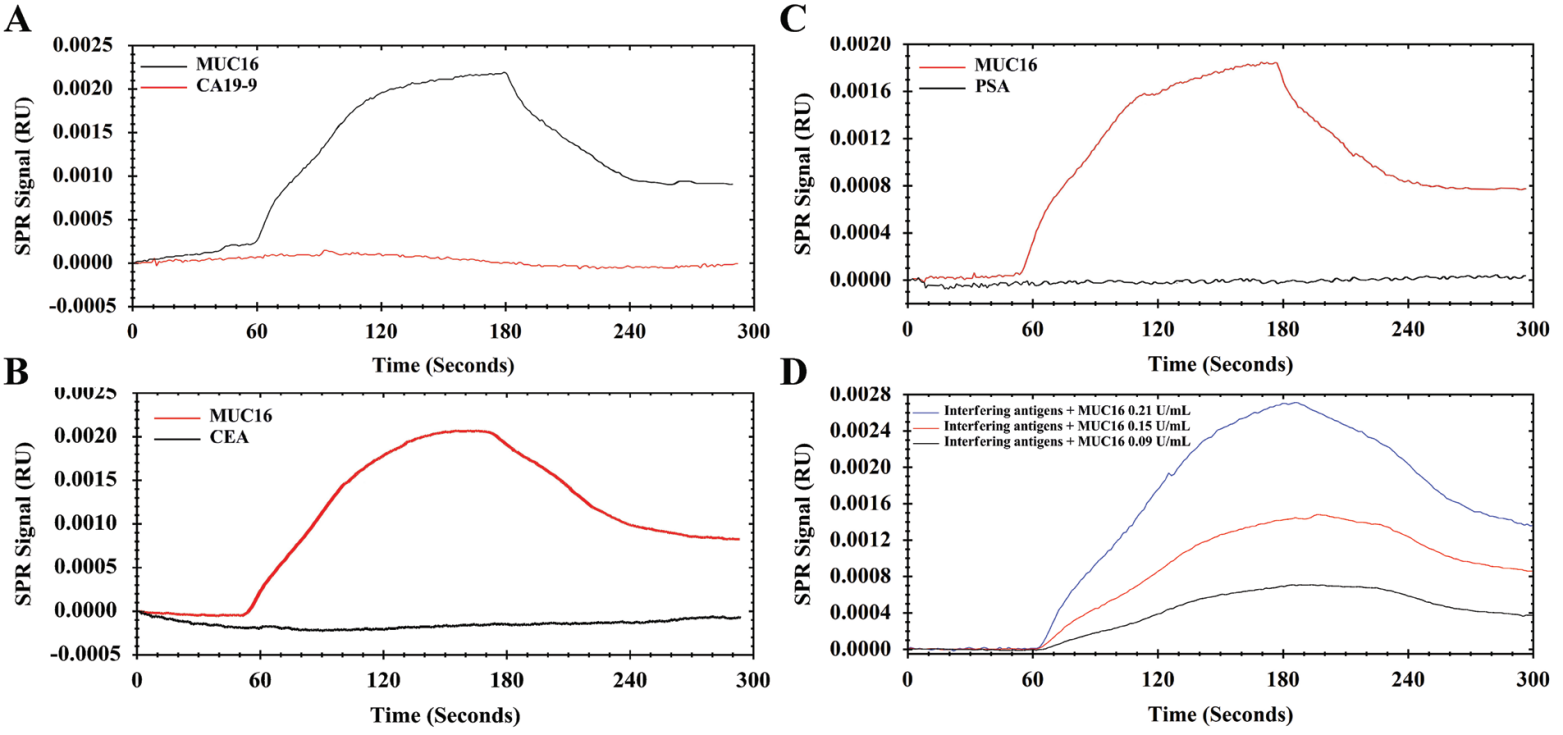
Sensograms show the specificity of the developed aptasensor toward different interfering proteins in optimized conditions (pH 7.2; T 25 °C). A) MUC16 (0.15 U/mL) versus CA19-9 (100 U/mL), B) MUC16 (0.15 U/mL) versus CEA (100 U/mL), C) MUC16 (0.15 U/mL) versus PSA (100 U/mL), and D) Spiked different concentration (0.09-0.21 U/mL) of MUC16 in mixed interfering antigens

 To demonstrate the selectivity of the Ap toward MUC16, the interference of different tumor markers (e.g*.,* CEA, PSA, and CA19-9) on the MUC16 response was investigated. The selection of interfering compounds was based on the validation characteristics of the commercial ELISA kits which are recommended for determination of MUC16. A solution of MUC16 (20 µL, 0.15 U/mL) and CA19-9 (20 µL, 100 U/mL) was introduced onto channel 1 and channel 2 simultaneously. As shown in Figure 5A, MUC16 gives a response of 0.0008 RU, which is much higher compared to CA19-9 with RU near the baseline (*P* < 0.0001). For differentiation and measurement of MUC16 in the presence of CEA (20 µL, 100 U/mL) as a negative control biomarker, the same injection was carried out. The results demonstrated significantly lower responses compared to MUC16 (Figure 5B). Besides, a similar test was performed to compare MUC16 and PSA antigen. The results showed that the PSA antigen did not influence the interaction of MUC16/Aps (Figure 5C). Additionally, the specificity of the aptasensor was confirmed by introducing the Ap with a mixture of MUC16 and mentioned interfering antigens. MUC16 mixtures with different concentrations (20 µL; 0.09, 0.15, 0.21 U/mL) spiked with interfering compounds (100 U/mL each of them) were tested. As shown in Figure 5D, a significant RU difference was found indicating that the PSA, CEA, and CA19-9 did not influence the quantification of MUC16. Therefore, this finding confirmed the specificity of the Ap surface towards MUC16, thus validating the high selectivity of the aptasensor.

###  Accuracy, precision, and recovery of developed aptasensor

 Quantification with different MUC16 concentrations (0.09, 0.15, 0.21, and 0.27 U/mL) was triplicated to determine accuracy, precision, and recovery following the ICH guideline. The accuracy was determined by the percentage of bias and should be less than 15% except at LOQ ( < 20%). Also, precision expressed by the percentage of RSD should be less than 15% (except at LOQ).^53^ Table 3 shows that both precision (-3 to –7%) and accuracy (2.7–11%) were lower than 15% for each concentration, and the recoveries of measurements were found to be in the range of 93%–96%. Therefore, based on the triplicated quantification results, it can be concluded that the precision, accuracy, and recovery for these measurements are satisfactory.

**Table 3:** Accuracy, precision, and recovery of the developed aptasensor quantified in standard samples

**True value (U/mL)**	**Measured value (U/mL)** **(mean±SD)**	**Bias (%)**	**RSD (%)**	**Recovery (%)**
0.09	0.085 ± 0.0094	-5	11	94
0.15	0.014 ± 0.0012	-6	8.6	93
0.21	0.195 ± 0.0072	-7	3.7	93
0.27	0.26 ± 0.007	-3	2.7	96

###  Repeatability and stability

 Three measurements (each measurement × 3) were performed every 2 hours (×3) to monitor the stability and repeatability of the developed biosensor. The bioanalytical method validation guideline specifies that stability should be less than 15%, and repeatability should be less than 15% of the standard deviation of short-time measurement.^54^ The stability and repeatability results for each concentration were < 9% and < 10%, respectively. Therefore, the stability and repeatability of the developed aptasensor were satisfactory for these measurements.

###  Matrix effect

 To study the matrix effect, the capability of the aptasensor was assessed to measure MUC16 in spiked MUC16 human serum samples. In this context, diluted human serum samples (1% v/v; diluted in phosphate buffer) were spiked with different concentrations of MUC16 (0.09 to 0.27 U/mL) to prepare spiked samples of MUC16, and then quantified using developed aptasensor. The experiments were conducted in optimized pH 7.2 and temperature 25°C conditions using spiked human serum samples at a 10 μL/min flow rate (each measurement × 3). Subsequently, the calibration curve was plotted and compared with the corresponding standard calibration curves (Figure 4), allowing for a comprehensive analysis of the results. The aim of the matrix effect experiment was to demonstrate the performance of the aptasensor in human serum samples, and also to investigate the sensitivity of the aptasensor in the presence of interfering components. The calibration curve slopes of both spiked and standard samples at pH 7.2 and temperature 25 °C showed no significant difference (*P >*0.05). The results of the triplicate measurement of spiked and standard samples (0.09 U/mL, 0.15 U/mL, 0.21 U/mL, and 0.27 U/mL) were compared and found to show no significant difference (*P* > 0.05). Besides, the obtained results in the matrix effect were compared with the results obtained by specificity data (Figure 5) and confirmed that the designed aptasensor was not sensitive to the matrix effect. Finally, the reliability of the developed aptasensor was evaluated by comparing the results of the designed aptasensor and a commercially available ELISA kit and ECL method (reference methods). For this, the results of the triplicate measurement were compared to the triplicate measurement reference methods. A significant difference was found between developed SPR technique and the two standard methods (*P* < 0.05).

 Compared to the developed SPR-based technique, reference techniques have several drawbacks, such as being time-consuming for the assay, requiring a high volume of sample, and often yielding negative or positive false results. The high sensitivity of the constructed SPR-based aptasensor can be attributed to the use of low-volume samples, which reduces matrix interference and improves detection precision. Furthermore, the SPR-based detection method is more straightforward and less prone to errors than many multistage diagnostic laboratory procedures.

## Conclusion

 In this study, a label-free aptasensor based on SPR was successfully developed to quantitatively measure MUC16 in real time. The anti-MUC16 Aps were immobilized onto the sensor chip using EDC/NHS chemistry under optimized conditions of pH 7.2 and temperature 25 °C. The kinetics and thermodynamics of the interaction between the Ap and MUC16 were investigated under these optimized conditions. The assay time for the aptasensor was only 6 minutes, demonstrating rapidity and efficiency. The biosensor had a linear range of 0.09 to 0.27 U/mL, with a LOD of 0.03 U/mL, indicating high sensitivity for the rapid screening of the MUC16 tumor marker. The recovery and precision levels of the aptasensor were satisfactory. The specificity of the aptasensor was evaluated by testing its performance in the presence of interfering biomolecules, and the results indicated that the developed aptasensor was highly specific. Additionally, the ability of the aptasensor to detect MUC16 in serum samples was investigated, and a detectable signal was observed with negligible difference in accuracy or precision. The biosensor’s performance was found to be comparable to currently used methods such as ECLIA and ELISA. In addition, the aptasensor exhibits a simpler design and operation in comparison to alternative methods commonly employed for the determination of MUC16.

## Acknowledgments

 We would like to express our gratitude to the Department of Biochemistry, Faculty of Biological Sciences, Tarbiat Modares University, and the Research Center for Pharmaceutical Nanotechnology at Tabriz University of Medical Sciences, Iran for providing us with both financial and technical assistance. We are truly thankful for their support and contribution to our research.

## Competing Interests

 The authors affirm that there are no competing interests or conflicts of interest related to this article.

## Ethical Approval

 Not applicable.
